# Synthesis of Ag_3_PO_4_/Ag/g-C_3_N_4_ Composite for Enhanced Photocatalytic Degradation of Methyl Orange

**DOI:** 10.3390/molecules28166082

**Published:** 2023-08-16

**Authors:** Qingwang Liu, Ying Meng, Qiman Liu, Mai Xu, Yunhu Hu, Shikun Chen

**Affiliations:** School of Chemistry and Materials Engineering, Huainan Normal University, Huainan 232038, China; qwliu2006@163.com (Q.L.); xumai2215@163.com (M.X.); huyunhu@ustc.edu.cn (Y.H.); 13956468746@163.com (S.C.)

**Keywords:** A_3_PO_4_/Ag/g-C_3_N_4_, photocatalyst, methyl orange

## Abstract

In this study, we have successfully constructed Ag_3_PO_4_/Ag/g-C_3_N_4_ heterojunctions via the hydrothermal method, which displays a wide photo-absorption range. The higher photocurrent intensity of Ag_3_PO_4_/Ag/g-C_3_N_4_ indicates that the separation efficiency of the photogenerated electron–hole pairs is higher than that of both Ag_3_PO_4_ and Ag/g-C_3_N_4_ pure substances. It is confirmed that the efficient separation of photogenerated electron–hole pairs is attributed to the heterojunction of the material. Under visible light irradiation, Ag_3_PO_4_/Ag/g-C_3_N_4_-1.6 can remove MO (~90%) at a higher rate than Ag_3_PO_4_ or Ag/g-C_3_N_4_. Its degradation rate is 0.04126 min^−1^, which is 4.23 and 6.53 times that of Ag/g-C_3_N_4_ and Ag_3_PO_4_, respectively. After five cycles of testing, the Ag_3_PO_4_/Ag/g-C_3_N_4_ photocatalyst still maintained high photocatalytic activity. The excellent photocatalysis of Ag_3_PO_4_/Ag/g-C_3_N_4_-1.6 under ultraviolet-visible light is due to the efficient separation of photogenerated carriers brought about by the construction of the Ag_3_PO_4_/Ag/g-C_3_N_4_ heterostructure. Additionally, Ag_3_PO_4_/Ag/g-C_3_N_4_ specimens can be easily recycled with high stability. The effects of hydroxyl and superoxide radicals on the degradation process of organic compounds were studied using electron paramagnetic resonance spectroscopy and radical quenching experiments. Therefore, the Ag_3_PO_4_/Ag/g-C_3_N_4_ composite can be used as an efficient and recyclable UV-vis spectrum-driven photocatalyst for the purification of organic pollutants.

## 1. Introduction

Semiconductor composite photocatalysis technology is a green, advanced, and efficient photocatalysis technology that deserves attention as a method for improving environmental pollution. Thus, the use of semiconductor-based novel visible light catalysts is of great concern [1,2,3,4].

In recent decades, photocatalysis experts have been committed to constructing new and efficient photocatalysts with narrow bandgaps to achieve a higher light conversion efficiency. In this regard, silver phosphate (Ag_3_PO_4_) is a promising photocatalyst, with a high photooxidation capacity, high quantum efficiency (90%), and low bandgap (2.5 eV) [5,6]. For this reason, many experts have reported on the photocatalytic performance of high-efficiency photocatalysts in the degradation of organic pollutants in water [7,8,9]. However, Ag_3_PO_4_ also has some drawbacks [10,11,12,13]. First, Ag_3_PO_4_ has a larger particle size and smaller specific surface area, which may affect its catalytic performance. Second, Ag_3_PO_4_ is not stable under long-term illumination, which hinders its recycling [14]. In addition, the micro-solubility of Ag_3_PO_4_ will reduce its structural stability. Therefore, it is a serious challenge to develop a highly efficient composite photocatalytic activity that maintains the stability of Ag_3_PO_4_ [15,16].

Graphite phase carbon nitride (g-C_3_N_4_) was first reported in 2009 and is a type of material with visible light activity, stable chemical properties, a cheap raw material price, and easy procurement. It is worth noting its unique layered structure and other advantages [17]. However, the pure g-C_3_N_4_ still has the problem of low separation efficiency of photogenerated charge carriers, resulting in an unsatisfactory photocatalytic activity.

The construction of a heterojunction is an effective way to solve the problem of the insufficient valence band oxidation ability of g-C_3_N_4_. Based on the original g-C_3_N_4_, another semiconductor with a strong valence band oxidation ability is introduced to combine it with. By regulating the interfacial charge directional migration, the light absorption and charge separation performance of g-C_3_N_4_-based materials can be optimized, while maintaining the strong oxidation and reduction ability of the system [18,19,20].

There are a few factors considered in the construction of a g-C_3_N_4_ heterojunction. The selected semiconductors are generally matched with appropriate band structures, and their connection methods make the best use of their unique properties. When considering the optimization of g-C_3_N_4_-based performance, it is critical to choose a suitable preparation method [21,22]. Studies have shown that the efficiency of photocatalysts greatly varies with the synthesis method. At present, there are numerous methods for preparing g-C_3_N_4_-based photocatalyst composites. The most original synthesis method is thermal polymerization, but the catalytic efficiency of g-C_3_N_4_ photocatalysts prepared by thermal polymerization is poor. Therefore, photocatalyst experts have developed a variety of synthesis methods. So far, although this has achieved remarkable results in the field of photocatalysis, there are still many key problems to be solved in the construction of efficient g-C_3_N_4_-based photocatalyst composites [23,24,25].

Recently, research on g-C_3_N_4_ and Ag_3_PO_4_ composite photocatalysts has been reported, suggesting that the g-C_3_N_4_/Ag_3_PO_4_ composite followed the double-charge-transfer mechanism. Inspired by this, it is a good strategy to use flake graphite phase g-C_3_N_4_ as a base to combine with Ag_3_PO_4_, which can compensate for the shortcomings in each semiconductor.

In this study, we successfully constructed a novel, efficient, and structurally stable photocatalyst for an Ag_3_PO_4_/Ag/g-C_3_N_4_ heterojunction through hydrothermal synthesis. Compared with Ag/g-C_3_N_4_ and Ag_3_PO_4_, the Ag_3_PO_4_/Ag/g-C_3_N_4_ composite photocatalyst showed more efficient photocatalytic activity for methyl orange degradation under visible light irradiation. In addition, the cyclic photocatalytic degradation of MO and its photocatalytic enhanced mechanism were investigated.

## 2. Results and Discussion

### 2.1. Structure and Morphology

**Figure 1:**
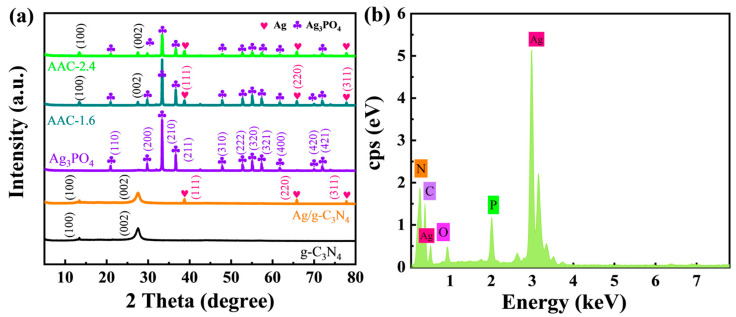
(**a**) XRD patterns of the different samples. (**b**) EDS pattern of the AAC-1.6.

Figure 1a shows the XRD patterns of the g-C_3_N_4_, Ag/g-C_3_N_4_, Ag_3_PO_4_, and Ag_3_PO_4_/Ag/g-C_3_N_4_ samples. For pure g-C_3_N_4_, there were two diffraction peaks at 13.26 and 27.4°. The main diffraction peaks at 13.2 and 27.4° were attributed to the (100) and (002) diffraction planes, respectively. For the Ag/g-C_3_N_4_ sample, the characteristic peaks in Figure 1a, at 38.4°, 65.8°, and 77.5°, marked with “
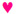
”, correspond to the (111), (200), and (311) crystal planes of metallic Ag. In the AAC-1.6 sample, Ag_3_PO_4_ is labeled by “
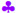
” [4,26]. The characteristic peaks of Ag, g-C_3_N_4_, and Ag_3_PO_4_ can be seen in Figure 1a, indicating that there were no other peaks in the sample, which contains only Ag, g-C_3_N_4_, and Ag_3_PO_4_. This indicates the high purity of the AAC-1.6 sample. Moreover, it can be seen from the EDS (energy-dispersive X-ray spectrometry) spectrum (Figure 1b) that the AAC-1.6 composite is composed of Ag, C, N, O, and P elements. The above suggests that the high purity of the AAC-1.6 sample is very beneficial for subsequent photocatalytic degradation experiments.

**Figure 2:**
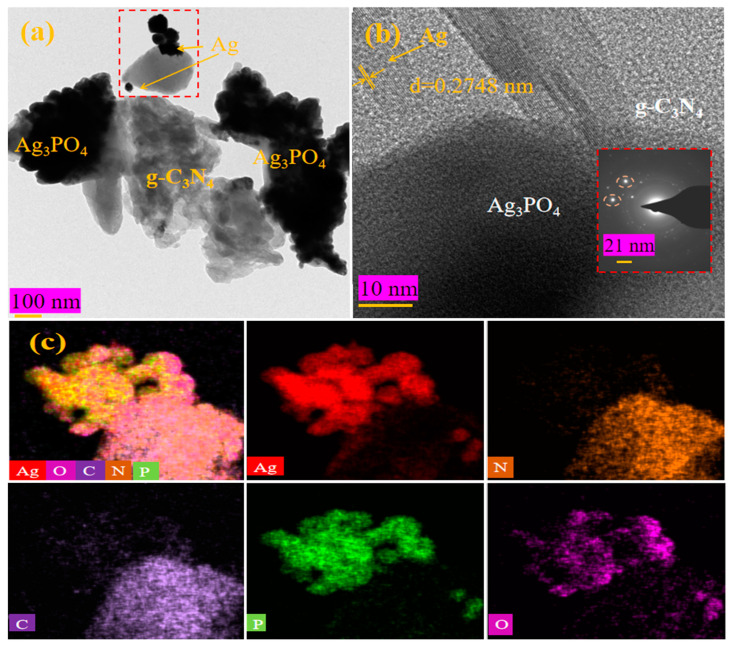
(**a**,**b**) TEM images of AAC-1.6. (**c**) EDX elemental mapping images of the AAC-1.6.

As shown in Figure 2a,b, Ag_3_PO_4_ particles adhered to g-C_3_N_4_ sheets, with effective physical contact between them. Ag particles were also seen attached to the interface between the two pure substances, g-C_3_N_4_ and Ag_3_PO_4_, with the presence of Ag mediating the electron transfer between them. Figure 2c shows the EDX elemental mapping image of all elements in the AAC-1.6 sample, with peaks of C, N, O, P, and Ag observed and labeled in the graph.

**Figure 3:**
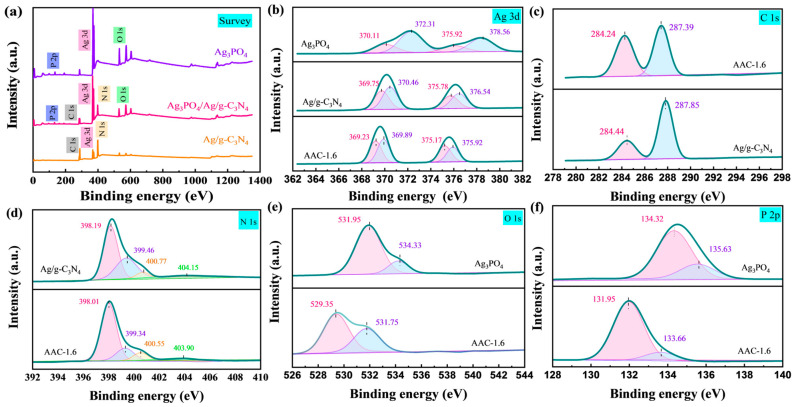
(**a**) XPS survey spectra of different materials: (**b**) Ag 3d, (**c**) C 1s, (**d**) N 1s, (**e**) O 1s, and (**f**) P 2p XPS spectra of AAC.

XPS (X-ray photoelectron spectroscopy) was used to study the composition and valence states of Ag_3_PO_4_, Ag/g-C_3_N_4_, and AAC-1.6 samples. As shown in Figure 3a, the survey scan XPS spectra of AAC-1.6 showed the presence of Ag, P, C, N, and O. The spectra of Ag_3_PO_4_ samples showed obvious peaks at 134.43, 533.72, and 372.44 eV, which were attributed to P 2p, O 1s, and Ag 3d, respectively. The Ag/g-C_3_N_4_ sample showed obvious peaks at 288.63, 398.12, and 372.43 eV, which were consistent with C 1s, N 1s, and Ag 3d. It is worth noting that the survey spectrum of AAC-1.6 powders was the same as the peaks of Ag_3_PO_4_ and Ag/g-C_3_N_4_ samples (134.44, 288.62, 372.45, 398.13, and 533.74 eV), indicating the presence of P, C, N, O, and Ag elements.

To further investigate the chemical valence states of the elements Ag, P, O, C, and N, high-resolution XPS patterns of Ag, P, O, C, and N were analyzed. The Ag 3d XPS pattern of Ag/g-C_3_N_4_ showed four major peaks (Figure 3b). The peaks with binding energies at 369.75 and 375.78 eV were attributed to the Ag 3d 5/2 and Ag 3d 3/2 spectra of Ag^+^ in Ag_3_PO_4_, and other peaks located at 370.46 and 376.54 eV could be attributed to metallic Ag [27,28,29,30,31]. Of interest, the XPS pattern of Ag 3d in AAC-1.6 showed a red shift of about 0.52 eV in the binding energy (369.23, 369.89, 375.17, and 375.92 eV) compared to Ag/g-C_3_N_4_, confirming some electronic interaction between Ag/g-C_3_N_4_ and Ag_3_PO_4_ in the composite.

However, the C 1s and N 1s spectra of AAC-1.6 did not change much from the binding energy of Ag/g-C_3_N_4_, which was only about 0.02 eV (Figure 3c,d). Due to the high content of Ag/g-C_3_N_4_ in AAC-1.6, no large binding energy shifts of C and N elements occurred.

It can be seen from the XPS pattern of the AAC-16 in Figure 3e that O 1s had two obvious peaks (529.35 and 531.75 eV), with the peak at 529.35 eV being attributed to the oxygen atom of silver phosphate, and the weak shoulder peak at 531.75 eV may be attributed to the terminal hydroxyl group (-OH) on the surface.

Subsequently, the P 2p XPS patterns of Ag_3_PO_4_ and AAC-1.6 samples were studied (Figure 3f). From Ag_3_PO_4_, it can be seen that there were two distinct peaks (134.32 and 135.63 eV) attributed to P 2p3/2 and P 2p1/2, respectively, corresponding to the phosphorus anion in the Ag_3_PO_4_ lattice [32,33,34]. Compared to Ag_3_PO_4_, the spectrum of P 2p in AAC-1.6 had two distinct peaks at 131.95 and 133.66 eV, with a lower band-edge energy, which was caused by a change in the chemical environment. Based on the above analysis, the AAC-1.6 composite formed the heterostructure.

### 2.2. Photoelectric Properties of Samples

**Figure 4:**
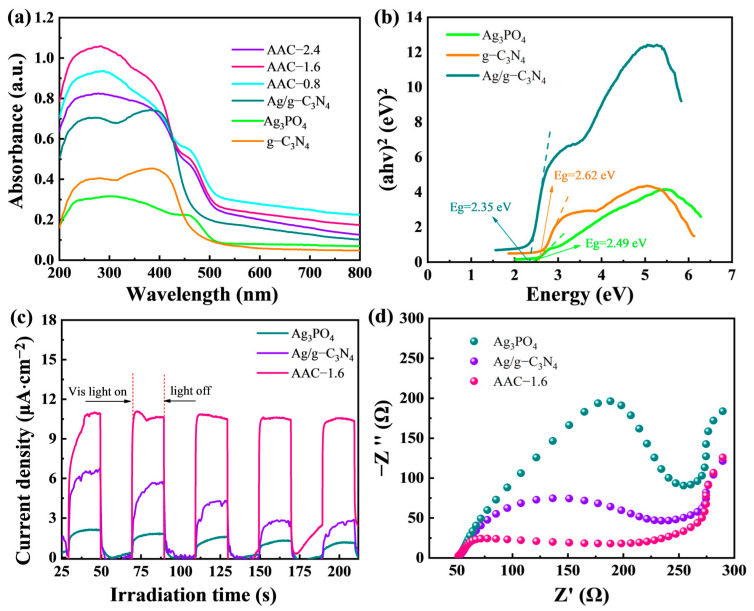
(**a**) The DRS spectra and (**b**) the calculated bandgap energies diagram of Ag_3_PO_4_, Ag/g-C_3_N_4_, and AAC-1.6. (**c**) Photocurrent response curves. (**d**) EIS Nyquist plots of Ag_3_PO_4_, Ag/g-C_3_N_4_, and AAC-1.6.

To investigate the light absorption capability of the AAC-1.6 nanocomposites, the DRS spectra were tested, and the results are shown in Figure 4a.

Pure Ag_3_PO_4_ absorbed in both the UV and visible regions, with most of the absorption concentrated in the UV region, and the sample with AAC-1.6 had the strongest absorbance. Strangely, the absorbance of the sample with AAC-2.4 was, on the contrary, lower than that of AAC-0.8, which was probably due to the excessive addition of g-C_3_N_4_ to block the light. In addition, several groups of samples showed poor absorption of the incident light when the incident light wavelength was greater than 500 nm. The AAC-1.6 sample exhibited enhanced absorbance due to the heterogeneous coupling of Ag_3_PO_4_ with Ag/g-C_3_N_4_, which showed enhanced absorbance in both the UV and visible regions, especially in the visible region, where the absorption intensity was significantly enhanced. Figure 4b shows the bandgap energy (Eg = E_VB_−E_CB_) transferred from the Kubelka–Munk function. According to the relationship between (αhν)^2^ and (hν), as shown in Figure 4b, the bandgaps of g-C_3_N_4_, Ag/g-C_3_N_4_, and Ag_3_PO_4_ were 2.62, 2.35, and 2.49 eV, respectively.

To analyze the performance of the photogenerated electron–hole pair separation, photocurrent response curves of Ag/g-C_3_N_4_, Ag_3_PO_4_, and AAC-1.6 samples were performed for five on/off irradiations under visible light (Figure 4c). As can be seen from Figure 4c, the strongest photocurrent density of 7.25 μA cm^−2^ was produced by Ag/g-C_3_N_4_ when the visible light was turned on. It is notable that the photocurrent density of AAC-1.6 jumped to 11.20 μA·cm^−2^ with continued light exposure, which is 1.5 times higher than Ag/g-C_3_N_4_ (7.25 μA·cm^−2^) and 7.0 times higher than Ag_3_PO_4_ (1.60 μA·cm^−2^). In addition, the electron transfer rate of the catalysts was determined by electrochemical impedance spectroscopy (EIS). As shown in Figure 4d, the EIS Nyquist plots of the three sets of samples showed different arc radii at high frequencies, and the smaller arc radii indicate a smaller transfer resistance at the electrode interface. The presence of a p-n-type heterojunction led to a smaller arc radius in the AAC-1.6 samples than in Ag/g-C_3_N_4_ and Ag_3_PO_4_, confirming that AAC-1.6 exhibited a lower photogenerated carrier transfer resistance than Ag/g-C_3_N_4_ and Ag_3_PO_4_. The above analysis indicates that enhanced charge transfer occurred in AAC-1.6, which contributed to the catalytic activity of AAC photocatalysts.

### 2.3. Photocatalytic Activity Test

**Figure 5:**
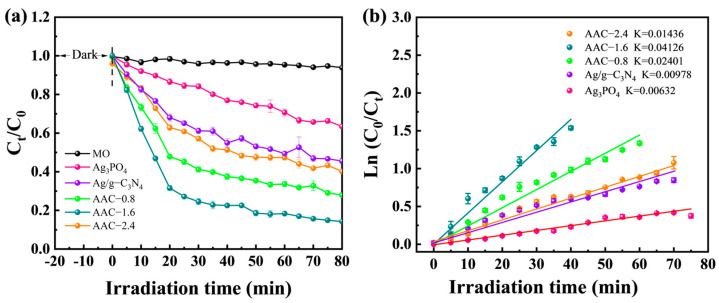
(**a**) Degradation curves for MO and (**b**) the corresponding first-order kinetics of the samples.

The photocatalytic activities of the prepared AAC composites were investigated through degrading MO dye in water phase. As shown in Figure 5a, all the prepared catalyst samples exhibited different excellent photodegradation performances in degrading MO, under 500 W xenon light irradiation. Worthy of attention, the AAC samples exhibited superior photocatalytic degradation efficiency compared to Ag_3_PO_4_ and Ag/g-C_3_N_4_, and the AAC-1.6 sample could degrade 99% of methyl orange in 80 min. Excessive Ag/g-C_3_N_4_ incorporation hindered the degradation rate. It can be seen from Figure 5b that the degradation rate constant of the AAC-2.4 sample was lower than that of AAC-0.8 and AAC-1.6. This might be because excessive Ag/g-C_3_N_4_ particles will block the contact between Ag_3_PO_4_ and incident light, leading to a decrease in the degradation efficiency, which is counterproductive to the photocatalytic performance of the AAC-2.4 photocatalyst. Due to the relatively low concentration of MO dye (37 mg·L^−1^), the quasi-primary kinetic reaction simulation behavior was met. The plots of ln (C_0_/C_t_) as a function of the irradiation time to MO are shown in Figure 5b. It can also be seen that AAC-1.6 had the highest degradation speed. The above results indicate that the efficient degradation efficiency of the AAC-1.6 composite material is attributed to the formation of heterojunctions, thereby enhancing the efficiency of photogenerated carrier separation [35,36].

### 2.4. Enhancement Mechanism and Stability

**Figure 6:**
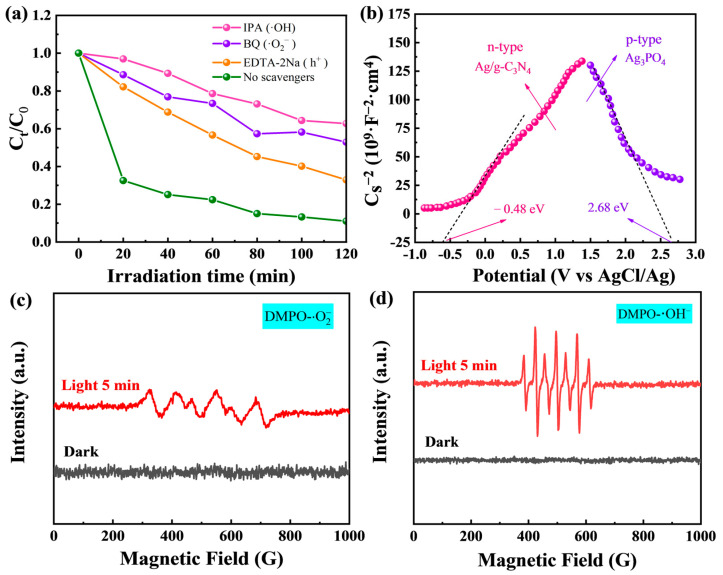
(**a**) Photocatalytic degradation of AAC-1.6 by free radical capture. (**b**) Mott–Schottky curves of samples. EPR spectra trapped by DMPO in the dark and under 5 min irradiation of visible light: (**c**) in methanol dispersion for DMPO- ${\cdot O}_{2}^{-}$ and (**d**) in aqueous dispersion for DMPO- ·OH of AAC-1.6.

For a more in-depth study of catalytically active species in photocatalytic reaction systems, a series of photocatalytic experiments with and without trapping agents were performed (Figure 6a). Three different scavengers, benzoquinone (BQ), ethylene diamine tetraacetic acid disodium salt (EDTA-2Na), and isopropanol (IPA), as trapping agents, were added to the MO solution to capture the corresponding active species (${\cdot O}_{2}^{-}$, h^+^, and ·OH). When IPA, BQ, and EDTA-2Na were added as scavengers, the degradation rate of MO decreased from 99% to 37.2%, 47.1%, and 67.1%, respectively. The capture experiments showed that ${\cdot O}_{2}^{-}$, ·OH, and h^+^ were effective for photocatalytic degradation in the photocatalytic reaction system and were the main active species in the catalytic reaction.

Besides, the semiconductor types and band-edge positions of Ag/g-C_3_N_4_ and Ag_3_PO_4_ were investigated by electrochemical Mott–Schottky curves in the dark state (Figure 6b). Ag/g-C_3_N_4_ is positively sloped and is an n-type semiconductor, with an estimated CB flat-band potential of −0.48 eV. On the contrary, the slope of Ag_3_PO_4_ is negative, indicating that it is p-type. The linear extension of two curves intersects the x-axis at two points, and the flat-band potential can be determined based on the horizontal coordinate values of the two points. The flat-band potentials of this intersection were estimated to be −0.48 and 2.68 eV, respectively. Generally, the conduction band potential of n-type semiconductors is about 0.2 V (vs. NHE ) less than the flat-band potential, and the valence band potential of p-type semiconductors is about 0.2 V (vs. NHE) more than the flat-band potential [37,38]. According to the equations (Eg = E_VB_−E_CB_) and E_VB_ = X−4.5 + 0.5E, X is the absolute electronegativity used to calculate the other values. Besides, the values of the flat-band potential, the CB potential of Ag_3_PO_4_, and the VB potential of Ag/g-C_3_N_4_ can be calculated to be 0.39 and 1.67 eV, respectively. For Ag_3_PO_4_-VB and Ag/g-C_3_N_4_- CB, they were calculated to be 2.88 and −0.68 eV, respectively. In addition, the authors of [4] showed that g-C_3_N_4_-VB and g-C_3_N_4_-CB are 1.53 and −1.09 eV, respectively. It is worth noting that the Mott–Schottky plot of AAC-1.6 showed a reverse **“**V**”**-shaped profile, indicating the expected p-n heterojunction.

EPR experiments on AAC-1.6 were examined under two different experimental conditions, visible light irradiation and darkness, and the results confirmed the production of hydroxyl radicals and superoxide radicals (Figure 6c,d). No signals of DMPO- ${\cdot O}_{2}^{-}$ and DMPO- ·OH were detected under dark conditions, indicating that there was no production of ${\cdot O}_{2}^{-}$ and ·OH under dark conditions [39,40]. Under visible light irradiation, the characteristic signals of DMPO- ${\cdot O}_{2}^{-}$ with an intensity ratio of 1:1:1:1 and DMPO- ·OH with an intensity ratio of 1:2:1:2:1 could be detected. As the illumination time increased to 5 min, the characteristic peak intensity of ${\cdot O}_{2}^{-}$ and the hydroxyl radical increased. The above results indicate that the ${\cdot O}_{2}^{-}$ and ·OH radicals produced by the sample AAC-1.6 play a crucial role in the catalytic degradation process.

**Figure 7:**
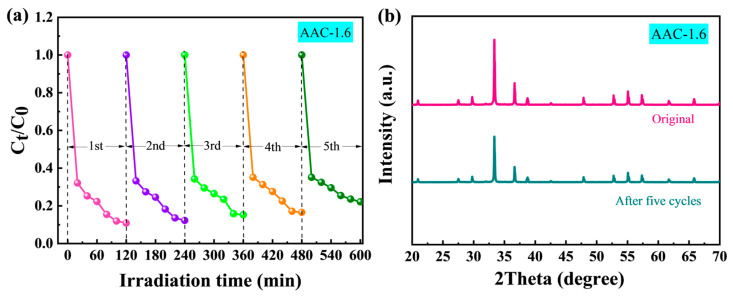
(**a**) Five degradation cycle curves of the photocatalyst for MO. (**b**) XRD patterns of AAC-1.6 before and after five cycles of degradation for MO.

As shown in Figure 7a, after 5 cycles, the degradation efficiency of MO by AAC-1.6 samples remained above 90%, indicating that AAC-1.6 samples had high stability in the degradation of MO. Subsequently, the XRD pattern spectra of AAC-1.6 powder before and after degradation were measured (Figure 7b). The AAC-1.6 powder exhibited a similar XRD pattern compared with the sample before degradation, reflecting that the AAC-1.6 powder had excellent stability.

**Figure 8:**
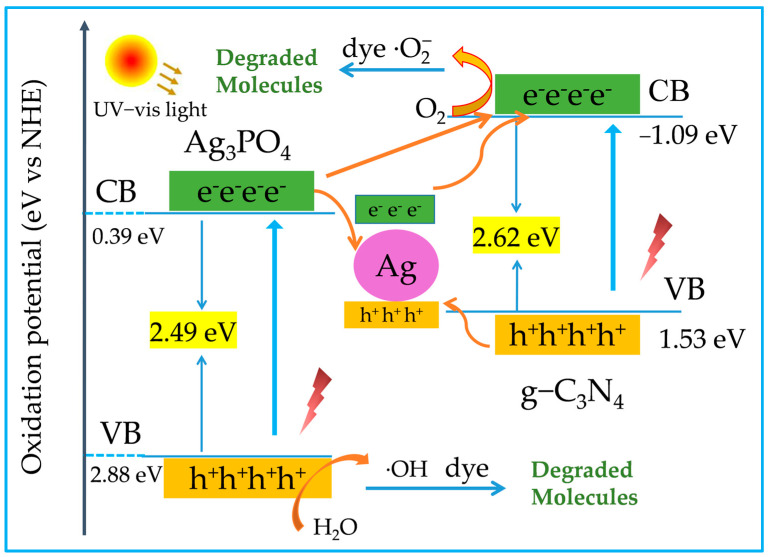
Schematic drawing of the proposed photocatalytic mechanism for p-n-type AAC composites under UV-visible light irradiation.

Based on the above discussion, the possible catalytic mechanism of the AAC composites is shown in Figure 8. when visible light irradiates the AAC composite catalyst material, electrons in the valence band (VB) of g-C_3_N_4_ and Ag_3_PO_4_ can be excited into their conduction band (CB) [41]. Similarly, metal silver can also absorb visible light, and a small portion of Ag nanoparticles (NPs) generate hot electrons through the plasma resonance effect. Hot electrons are transferred from the surface of Ag NPs to the conduction band (CB) of the pure substance g-C_3_N_4_. The electrons on g-C_3_N_4_-VB can convert O_2_ into superoxide radicals to degrade pollutants, while the holes are still retained in the valence band of Ag nanoparticles. Meanwhile, the photogenerated electrons in the conduction band of Ag_3_PO_4_ are transferred to the surface of Ag nanoparticles to combine with plasma resonance-induced holes, and the photogenerated holes of pure Ag_3_PO_4_ remain on Ag_3_PO_4_-VB. The holes (h^+^) on Ag_3_PO_4_-VB oxidize H_2_O to generate ·OH radicals for the degradation of organic pollutants [42,43].

## 3. Materials and Methods

### 3.1. Materials

Silver nitrate, polyvinyl pyrrolidone, melamine, and the experimental reagents (Na_2_HPO_4_·12H_2_O and MO) were provided by Sinopharm Chemical Reagent Co., Shanghai Branch, Shanghai, China [44]. All experimental reagents used were analytically pure and solutions were prepared with ultrapure or deionized water.

### 3.2. Synthesis of Samples

#### 3.2.1. Preparation of Ag/g-C_3_N_4_

The bulk g-C_3_N_4_ was synthesized by heating melamine (25 g, 39.60 mmol) in air at a temperature of 500 °C for 6 h, with a heating rate of 5 °C per minute. After calcination, the yellow powder sample g-C_3_N_4_ was obtained by grinding bulk g-C_3_N_4_. Typically, g-C_3_N_4_ (2.4 g, 26.10 mmol) was added to an 80 mL methanol solution, and suspension A was obtained after ultrasonication for one hour. A solution of AgNO_3_ in water (0.24 mol·L^−1^) was added dropwise to suspension A, and stirred at 70 °C for two hours. Simultaneously, the suspension was exposed to irradiation by a 500 W Xe lamp for two hours [45,46]. Then, the target sample g-C_3_N_4_ (the average particle size of g-C_3_N_4_ is 8 μm) was obtained by filtration, washing, constant temperature (70 °C), and vacuum drying for 10 h.

#### 3.2.2. Preparation of Ag_3_PO_4_/Ag/g-C_3_N_4_

Typically, 0.4 g Ag/g-C_3_N_4_ was added to a 50 mL methanol solution, and solution A was obtained after ultrasonication for one hour. Then, 0.4890 g of AgNO_3_ and 1.5012 g of polyvinyl pyrrolidone were added to 60 mL of ultrapure water, and solution B was obtained. Then, solution B was directly added to solution A, and Na_2_HPO_4_·12H_2_O solution was (0.08 mol·L^−1^) added drop-by-drop to the mixed solution with a constant-pressure drop funnel. The mixed solution reacted for one hour with stirring. Next, the samples were washed three times with deionized water and three times with ethanol, and then dried in a drying oven (80 °C under vacuum for 10 h) [47]. The resulting samples were simply denoted as AAC-x, with x representing the molar ratio of different Ag_3_PO_4_ to Ag/g-C_3_N_4_ (x = 0.8, 1.6, 2.4) [48,49].

### 3.3. Analytical Characterization

The samples’ crystal structure was measured by a Bruker D8 Advance diffractometer with CuKα (λ = 0.154056 nm) and a scanning range of 2θ = 5°~80° (XRD, BRUKER D8 ADVANCE, Massachusetts, Germany). The morphology and crystalline structure were observed with a FEI Talos-F200S scanning electron microscope (TEM, FEI Company, Hillsboro, OR, USA). The diffuse reflectance spectra were determined by DRS, with a UV-3700 UV-Vis Spectrometer (Shimadzu, Kyoto, Japan). The flat-band potential was measured by a three-electrode electrochemical workstation (Huachen, CHI660E, Shanghai, China). The chemical composition and element valence of the samples were detected by XPS (the K-alpha radiation of aluminum, X-ray photoelectron spectrometer, Thermo Fisher Scientific, ThermoScientific K-Alpha, Waltham, MA, USA), and the charge shift was corrected by the C 1s peak of the contaminated carbon (284.8 eV). The free radicals formed in the photocatalytic process were identified by an EPR (electron paramagnetic resonance) spectrometer (Bruker EMXplus-6/1, Massachusetts, Germany). Photocatalytic testing was performed using the ZQ-GHX-V (Shanghai Zhengqiao Scientific Instruments Co., Ltd., Shanghai, China).

### 3.4. Measurement of Photocatalytic Activity

To achieve adsorption equilibrium between the photocatalyst and the dyes, 0.1 g of each catalyst was added to a 50 mL solution (1.0 × 10**^−^**^5^ mol·L**^−^**^1^) containing methyl orange (MO), and the mixture was stirred away from light for one hour. Subsequently, the dye solution was subjected to catalytic degradation evaluation under a 500 W xenon lamp. During the photocatalytic process, about 5.0 mL of the solution was withdrawn every 5 min and centrifuged, and the absorbance of the supernatant was measured at the maximum absorption wavelength (505 nm) by UV-vis spectrometry, and then the data were recorded.

## 4. Conclusions

The AAC-1.6 photocatalytic composite material constructed by the hydrothermal method exhibited excellent degradation efficiency in the process of photocatalytic degradation of organic pollutants. The high efficiency of photocatalytic composite materials in degrading pollutants is attributed to the formation of heterojunctions. The experimental results indicated that heterojunctions exhibited higher charge separation and transfer efficiency, as well as a stronger redox ability, in the catalytic degradation process. In addition, the capture experiment results showed that superoxide radicals and hydroxyl radicals were the main active substances and played a key role in the photocatalytic degradation of methyl orange. This work is expected to provide an effective solution for the further development of efficient, narrow-bandgap photocatalysts, and it may provide new ideas for the construction of efficient composite photocatalysts, which is crucial to solve the problem of environmental water pollution.

## Data Availability

Not applicable.
